# Vacuum Erection Device Plus Once-Daily Tadalafil Improve Clinical Outcomes after Extracorporeal Shock Wave Therapy in Men Affected by Erectile Dysfunction Associated with Peyronie’s Disease

**DOI:** 10.3390/life14091162

**Published:** 2024-09-13

**Authors:** Lucio Dell’Atti, Viktoria Slyusar, Piero Ronchi, Chiara Cambise

**Affiliations:** 1 Department of Urology, University-Hospital of Marche, 60126 Ancona, Italy; 2 Pain Therapy Center, Division of Anesthesia and Intensive Care, University-Hospital of Marche, 60126 Ancona, Italy; 3 Department of Emergency, University-Hospital Gemelli IRCSS, 00168 Roma, Italy

**Keywords:** Peyronie’s disease, extracorporeal shockwave, vacuum erection device, erectile dysfunction, phosphodiesterase type 5 inhibitors

## Abstract

Background: The purpose of this study is to examine the combination of the mechanical effects of penile therapy with vacuum erection devices (VEDs) plus PDE5i, which improve clinical outcomes after extracorporeal shockwave therapy (ESWT) in men affected by erectile dysfunction (ED) associated with Peyronie’s disease (PD). Methods: A total of 153 medical records of patients affected by PD in stable stage with ED and treated with ESWT were divided into two groups. Group A (GA) included 72 men treated with ESWT, mechanical stretching with VEDs and PDE5ì (Tadalafil 5 mg), and Group B (GB) included 81 men who received only ESWT plus Tadalafil 5 mg with the same protocol of GA. The patients in both groups were assessed at baseline and follow-up for erectile function, painful erections, penile plaque size, and penile curvature. The results were evaluated at baseline and 3, 6, and 12 months after the treatments. Results: Three months after the treatment, GA patients had a reduction in penile curvature degree from a mean ± SD of 33.91 ± 8.34° at baseline to a mean ± SD of 19.46 ± 7.15° after 12 months, whereas pain in an erection or during intercourse was resolved completely in 88.9% of the patients. The mean ± SD IIEF-15 score of patients affected by severe/moderate ED further improved significantly in the GA group (*p* < 0.001) after 3, 6, and 12 months of treatment. There were no permanent adverse sequelae after treatments. Conclusions: The regular use of a VED plus Tadalafil in patients who had undergone ESWT significantly provided more benefit in patients with PD in terms of penile deformity, pain, and erectile function.

## 1. Introduction

Peyronie’s disease (PD) is an acquired idiopathic fibrotic degeneration affecting the tunica albuginea of the corpora cavernosa with a prevalence in men over 40 years ranging from 0.4% to 7% [1]. The causes are unknown, but the dominant theory concerns recurrent micro-trauma to the corpora cavernosa during sexual intercourse. PD stands out in an acute inflammatory phase (active stage) with the formation of one or more plaques followed by a chronic stage where the plaques become stables in a time ranging from 12 to 16 months with associated persistent pain [1,2]. Management of PD during the active stage includes conservative therapy such as oral drugs, topical agents, intra-plaque injections, mechanical stretching with vacuum erection devices (VED), and extracorporeal shockwave therapy (ESWT) [3]. Shock wave therapy has been utilized for a variety of conditions in medical settings. This technology uses acoustic wave energy to transmit pressure through a designated medium that can then be focused on a specific point and managed as a mechanical force to create changes in the biological tissues [4]. The treatment is a minimally invasive approach in that the mechanical stimulation of the waves on cellular tissues induces a “cavitation effect” with the generation of nitric oxide and vascular endothelial growth factor with a subsequent phase of neovascularization. Stretching VEDs are non-invasive options under study and may be a viable method of halting disease progression and possibly reversing pathological penile curvature [5,6]. Penile traction in PD is theorized to work by the mechanism where repeated traction extends the fibrinous plaque by reorganization of the extracellular collagen matrix. Besides physical symptoms, PD impacts sexual function, and it is associated with psycho-social distress for the patients and even for their partners [6]. Penile deformity could lead to pain, discomfort during penetrative intercourses, and erectile dysfunction (ED). The prevalence of ED in the PD population ranges from 40 to 60%. Individual therapeutic options are more complex in PD patients with ED when considering the treatment of both problems [7]. Phosphodiesterase type 5 inhibitors (PDE5i) have long been used in the treatment of ED. In PD, these agents are proposed to inhibit tissue remodeling after acute injuries by decreasing oxidative stress responsible for inflammation and fibrosis [8]. Moreover, these oral drugs are safe and effective and are considered first-line therapy for PD patients with ED [9]. The hypothesis of this study is that the combination of the mechanical effects of penile therapy with a VED plus PDE5i (Tadalafil) improves clinical outcomes after ESWT in men affected by ED associated with PD.

## 2. Patients and Methods

### 2.1. Study Design and Selection Criteria

Data were collected retrospectively for all PD patients referred between January 2018 and May 2023 at two referral andrology centers. The research was conducted according to the Declaration of Helsinki on ethical principles for medical research involving human subjects. The inclusion criteria were patients aged ≥18 years with PD lasting for ≥12 months, presence of only one stable penile plaque ≤ 20 mm in largest diameter, no medical treatment for the previous 3 months, penile deviation of ≤60°, associated ED. The exclusion criteria were presence of two or more penile plaques, prior penile surgery or ESWT, prior injection intraplaque therapy, low testosterone total (TT) serum levels (normal reference range: 300–1000 ng/dL), patients in PD active stage and in the presence of coagulopathy disease. Accurate sociodemographic data, medical history, and physical examination were obtained during the first visit. The following data were recorded: age, previous treatments, medications, comorbidities, penile trauma history, location, number and size of plaques, disease duration, type and degree of penile curvature, ED, and pain during erection. Size, number, and location of penile plaques were assessed (in mm) with an ultrasonography study (with a 7.5 to 12 MHz linear transducer). The degree of the penile curvature angle was measured with a goniometer after intracavernous vasoactive injection (alprostadil 10–20 mcg) before and after the treatment. ED was evaluated using the simplified International Index of Erectile Function (IIEF-15). Severity of ED was classified as severe (IIEF-15 ≤ 10), moderate (IIEF-15 between 11 and 16), or mild (IIEF-15 between 17 and 21). The subjective pain during an erection was evaluated by means of the visual analog scale (VAS), with 0 as no pain and 10 as maximum pain. The results were evaluated at baseline and 3, 6, 12 months after the treatment. Written informed consent was obtained from the eligible patients prior to each new treatment.

### 2.2. Treatment Protocols

The Duolith SD1 T-TOP URO (Storz Medical AG^®^, Tägerwilen, Switzerland) device was utilized for the treatment of patients by two expert urologists. The penis was stretched, and a water-based lubricant gel was put on plaque size to ensure optimal shock wave transmission. In every session, patients received a total of 3000 shocks. Waves were distributed with an incremental level of energy from 0.15 mJ/mm^2^ to 0.30 mJ/mm^2^ and a frequency of 6–8 Hz. Each session included 3000 shockwaves addressed to the major penile plaque. The complete treatment for patients with PD consisted of the sequential application of ESWT protocol over 6 weeks for each patient. All patients were treated without anesthesia in an outpatient setting and were monitored for adverse events such as pain, hematoma, or skin redness. After ESWT, patients were well informed regarding the benefits/risks of complementary associated treatment options (VED and/or PDE5i). The patients provide drugs and vacuum devices by themselves. Vacuum devices were proposed as a supplementary treatment option, and therefore, they were collected in two groups according to their intention: purchasing drugs only or devices plus drugs. Patients in Group A (GA) were instructed to use the VED for 10 min twice a day for 3 months, which was combined with Tadalafil 5 mg once daily. Patients in Group B (GB) were only treated with Tadalafil 5 mg once daily for 3 months. The VEDs (Medis, Medical Services, Milan, Italy) used in GA were manually operated.

### 2.3. Statistical Analysis

The data were analyzed in a common database. Continuous variables were described as medians and interquartile ranges and nominal variables were described as numbers and percentages. Comparisons between groups were performed using the Chi-square test or Fisher’s exact test for discrete variables and Mann–Whitney U test for continuous variables. All statistical analyses were conducted using SPSS version 24 (IBM Corp., Armonk, NY, USA).

## 3. Results

A total of 153 medical records of patients affected by PD with ED and treated with ESWT were included retrospectively in the study. Among them, GA was composed of 72 patients treated with ESWT, mechanical stretching with a VED and PDE5ì (Tadalafil 5 mg), and GB consisted of 81 patients that received only ESWT plus Tadalafil 5 mg with the same protocol of GA. Patient’s characteristics at inclusion are shown in Table 1. Statistically insignificant differences emerged between the two groups at baseline, except for higher presence of patients with ED in GA (61.1%) vs. GB (54.3%) (*p* < 0.001). A total of five patients, two patients in GA (2.8%) and three patients (3.7%) in GB, received surgical correction for their dissatisfaction at the end of the treatment. There were no clinical hematomas requiring intervention reported after treatments (Table 2). Six patients of GA (8.3%) and five of GB (6.2%) had a superficial hematoma at treatment site, but there were no permanent adverse sequelae. All patients underwent at least six treatment sessions. The median follow-up was 14.6 months. Three months after the treatment, the patients in both groups had an insignificant reduction in penile curvatures. However, GA patients had a clinically significant reduction in the curvature from a mean ± SD of 33.91 ± 8.34° at baseline to a mean ± SD of 19.46 ± 7.15° after 1 year (*p* < 0.001) by treatments, whereas erection and improvement in ability to perform intercourse occurred completely in 88.9% (64/72) of the patients. In GB, patients had a decreased curvature degree from a mean ± SD of 32.64 ± 8.87° at baseline to a mean ± SD of 24.10 ± 8.43° after 1 year (*p* < 0.001), whereas intercourses were improved in 60.5 % (49/81) of men. According to correlation analysis, there were no statistically significant differences between treatments and penile plaque sizes at 3 months after treatment in both groups. However, after 1 year (*p* = 0.001), particularly in the GA, significant differences were observed. The mean ± SD IIEF-15 score of patients affected by severe/moderate ED further improved significantly in the group of patients treated with ESWT and a VED plus PDE5i (*p* < 0.001) after the 3, 6, and 12 months of treatment (Table 3).

## 4. Discussion

The results of this study provide evidence that the regular use of a VED plus Tadalafil in a patient who had undergone ESWT significantly provided more benefit in patients affected by PD and ED in terms of penile deformity, pain, and erectile function.

Many treatments discussed above should provide greater benefit in patients with PD. However, these medical treatment options considered separately for PD are suboptimal and can leave patients with physical and psychological morbidity [2,3]. One of the biggest consequences of leaving PD untreated is ED. Some PD patients are unable to penetrate due to ED, painful erections, and/or the angle of the curvature. ED is present in 20–50% of PD patients and occurs for a variety of reasons, including penile curvature and cavernosal fibrosis, which compromise penile vascularity [7]. In our study group (GA), ED was reported as severe in 27% of men and moderate in 35% of men, while in the other group (GB), these percentages were 19% and 36%, respectively. Given our elderly patient population and the presence of comorbidities, it may be a contributing factor for ED. Metabolic and cardiovascular diseases with increased prevalence in the elderly could play a crucial role in the pathogenesis of many cases of age-related ED [10]. Although there are no statistically significant differences between the ages of the two groups, we note the presence of patients with diabetes and hypertension more in GB than in GA. A common link between ED and PD may also be hypogonadism. Indeed, TT deficiency is present in up to 60% of men with PD and is related to worse curvature compared to men with normal TT concentrations [11]. It is now standard practice to treat hypogonadal patients with testosterone replacement to improve their ED. Thirty patients who were excluded from the study reported low TT serum levels when they were diagnosed with PD. Recently, ESWT has been gaining interest in the treatment of PD and ED thanks to its mini-invasive characteristics [12]. Hauck et al. [13], in a meta-analysis, observed that a decrease in plaque size was reported in 0% to 68% of cases, a decrease in curvature was reported as providing no significant findings in 74% of cases, and a decrease in penile pain was reported in 56% to 100% of cases. The molecular mechanism of action has not been clearly defined; however, shock waves are used to disrupt the dense scar tissue. No improvement in penile curvature deformity or plaque size has been shown in placebo-controlled trials using ESWT. Compared to baseline, after ESWT, patients had no significant change in mean plaque size or mean penile curvature deformity [3,4]. There are several recent studies investigating lithotripsy as a treatment for men with ED unresponsive to PDE5is. These studies examining small numbers of patients show that this is a safe option with few complications [14,15]. The mechanisms underlying lithotripsy as a treatment option for ED are unclear but are thought to be associated with improvements in penile hemodynamics and endothelial function. In the randomized, double-blind, sham-controlled study conducted by Vardi et al., ESWT alone has been shown to have a beneficial effect on ED in men with PD [16]. Instead, an open-label single-arm prospective study by Gruenwald et al. observes that ESWT has a physiologic effect on the erectile mechanism and improves IIEF-ED [17]. The protocol was two treatment sessions per week for 3 weeks, which were repeated after a three-week no-treatment interval. The prospective, randomized, double-blind, placebo-controlled study by Palmieri reported that after 12 weeks, the VAS score and IIEF score ameliorated significantly after ESWT [18]. After 24 weeks, plaque size and curvature degree were significantly reduced. Indeed, Palmieri et al. evaluated that mean plaque size and mean curvature degree were significantly higher in the placebo group when compared with both baseline and ESWT values. Treatment sessions were performed once weekly for four consecutive weeks [18]. In agreement with the results in studies performed by Vardi et al. [16], Gruenwald et al. [17], and Palmieri et al. [18], we have found improvement in all the domains of IIEF and in the sexual function, reduction in VAS score, plaque size, and penile curvature. Regarding the VAS score, in our study, it was reduced by 5 points after treatment compared to baseline. To our knowledge, no previous studies examined the add-on effect of vacuum pump and PDE5i to ESWT on PD. Vacuum pump and ESWT are both considered conservative treatments with few side effects. Continuous traction devices, such as penile VEDs, employ their effect by increasing the activity of degrading enzymes. This initially causes loss of tissue strength, and the PD plaque becomes solvable and degraded [19]. The process is followed by an increase in newly synthesized collagen. In a randomized, single-blinded clinical trial noted after 6 months, the mean change in penile curvature was −12.8 (SD 13) degrees in the treatment group and −6.6 (SD 8.9) in the control group (*p* = 0.204). The mean IIEF-15 score decreased by 0.17 (SD 3) in the treatment group and 3.06 (SD 5.5) in the control group (*p* = 0.086) at the six-month follow-up. In the treatment group, the authors observed a greater but non-significant change in penile curvature [20]. ESWT has previously demonstrated an ability to improve vascularization, wound healing, angiogenesis. Tadalafil is also a unique PDE5i with a long stage of responsiveness [20,21]. It can improve erectile function by inhibiting the PDE5 isoenzyme, leading to blocking cGMP degradation. The PDE5i acts with NO and enhances the levels of cGMP, which, in the presence of sexual stimulation, leads to the erection [21]. In the current study, the degree of penis curvature and plaque size decreased after ESWT in the group with a VED and oral Tadalafil. Park et al. [22] reported that Tadalafil (5 mg once daily) could treat PD by reducing the degree of penile curvature and penile plaque size. Tadalafil may play the main role in stabilizing penis curvature and plaque fibrosis. Porst et al. [23] observed that Tadalafil in low doses (2.5 and 5 mg) was effective and well-tolerated. Moreover, Tadalafil can be administered due to longer half-time, and its use is associated with patient satisfaction. Palmieri et al. [24] used 5 mg Tadalafil and ESWT to treat patients with PD and observed significant improvement in sexual function and quality of life of men with ED and PD. The authors reported no significant difference between patients receiving ESWT alone and those receiving daily Tadalafil with ESWT regarding curvature degree, indicating no effect of Tadalafil on curvature degree. Moreover, neither ESWT alone nor Tadalafil with ESWT was able to improve plaque size [24]. All this appears in contrast to the analysis of our study, where significant differences emerged between baseline mean plaque size, penile curvature, VAS score, and IIEF-15 score in patients with PD receiving this therapeutic association. Recent studies show that high-energy shock wave therapy is safe and efficient and provides stable long-term results. Rassweiler et al. [25], in a retrospective non-randomized study, evaluated 85 patients for chronic phase Peyronie’s disease with large plaques [89.9 (2–450) mm^2^] using two electromagnetic lithotripters applying high-energy shock waves under local anesthesia and sonographic/fluoroscopic control. The authors observed that 76% of patients were free of pain and showed an improvement of penile curvature in 51%, with an improvement in sexual intercourse in 68% of patients. In our opinion, in cases with significant plaque formation, the concept of high-energy ESWT should be considered in future studies, particularly when large plaques are present.

However, our study has several limitations. It was a non-randomized and retrospective study. This study was conducted by two experienced urologists at two referral andrology centers. The grouping procedure reflected the choices of patients; considering the cost of vacuum devices plus PDE5i, it is possible that the cost of purchasing the device represented a bias factor. Patients who are richer or more motivated could afford to buy the device, and therefore, this could lead to a selection bias. Furthermore, the two groups present some significant differences that should be considered potential bias factors. Patients in group A were more frequently on PDE5i at the start of treatment (25% vs. 17.3%). Longer treatment with PDE5i on-demand could favor this group, and this aspect also includes a bias. As a final point, the rate of severe ED in group A is a significant limitation, and these patients could be more motivated. The lack of long-term outcomes must be validated with future multicenter studies.

## 5. Conclusions

We showed that VED combination therapy could achieve extra benefits in patients who had undergone ESWT. The addition of VED to regular use of Tadalafil significantly reduced their subjective concerns associated with ED and provided more benefit in patients with PD in terms of penile deformity, pain, discomfort during penetrative intercourses, and erectile function.

## Figures and Tables

**Table 1:** Patient’s characteristics.

Patients’ Characteristics before Treatments	Group A (ESWT, VED, PDE5i) *n*: 72	Group B (ESWT, PDE5i) *n*: 81	*p* Value
Age (years)	51.6 (28.4–66.9)	54.4 (31.6–65.7)	NS
Duration of disease (months)	13.6 (12.4–13.9)	13.9 (12.6–14.5)	NS
Smoking, *n* (%)	28 (38.8)	33 (40.7)	NS
Diabetes, *n* (%)	5 (6.9)	8 (9.8)	NS
Hypertension, *n* (%)	8 (11.1)	10 (12.3)	NS
Dupuytren’s disease, *n* (%)	0	1(1.2)	NS
ED (IIEF-15 score), *n* (%)			
severe (IIEF-15 ≤ 10),	19 (26.4)	15 (18.5)	<0.001
moderate (IIEF-15 between 11 and 16)	25 (34.7)	29 (35.8)	NS
mild (IIEF-15 between 17 and 21).	28 (38.9)	36 (44.4)	<0.001
Plaques size, (mm)	12.31 ± 7.58	12.55 ± 6.97	NS
Sonographic findings, *n* (%)			
Hypoechoic	29 (40.3)	34 (41.9)	NS
Hyperechoic	33 (45.8)	36 (44.5)	NS
Mixed	10 (13.9)	11 (13.6)	NS
Plaque position, *n* (%)			
Ventral	12 (16.9)	14 (17.3)	NS
Dorsal	33 (46.5)	36 (44.4)	NS
Lateral	26 (36.6)	31 (38.3)	NS
Treatments, *n* (%)			
Vitamin E	22 (30.5)	27 (33.3)	NS
Nutraceutical Drugs	31 (43.1)	38 (46.9)	NS
PDE5i (on-demand)	18 (25)	14 (17.3)	<0.001
Alprostadil	1 (1.4)	2 (2.5)	NS

ESWT: extracorporeal shockwave therapy; VEDs: vacuum erection devices; PDE5i: Phosphodiesterase type 5 inhibitors; NS: not significant; ED: erectile dysfunction; IIEF: International Index of Erectile Function.

**Table 2:** Adverse events reported after the treatments.

Adverse Events (*n*, %)	Group A *n*: 72	Group B *n*: 81	*p* Value
Hematoma	6 (8.3)	5 (6.2)	NS
Pain	2 (2.7)	3 (3.7)	NS
Skin flushing	5 (6.9)	6 (7.4)	NS
Neurapraxia	1 (1.4)	1 (1.2)	NS
Other events	-	-	-

NS: not significant.

**Table 3:** Comparison of clinical outcomes between two groups of patients before and after ESWT.

Clinical Outcomes after Treatments	Time	Group A (ESWT, VED, PDE5i) *n*: 72	Group B (ESWT, PDE5i) *n*: 81	*p* Value
Plaque size (mm)	Before	12.21 ± 7.33	12.89 ± 7.82	NS
	After 3 months	10.33 ± 7.89	10.45 ± 7.11	NS
	After 6 months	07.22 ± 6.59	10.72 ± 6.23	0.001
	After 12 months	07.13 ± 5.77	10.68 ± 6.85	0.001
* p * *		<0.001	NS	
Penile curvature (°)	Before	33.91 ± 8.34	32.64 ± 8.87	NS
	After 3 months	30.35 ± 7.22	31.14 ± 8.31	NS
	After 6 months	25.13 ± 8.64	28.42 ± 7.59	0.001
	After 12 months	19.46 ± 7.15	24.10 ± 8.43	0.001
* p * *		<0.001	<0.001	
Pain during erection (VAS score)	Before	6.53 ± 1.82	6.63 ± 1.89	NS
	After 3 months	2.11 ± 1.22	2.79 ± 1.63	NS
	After 6 months	1.69 ± 1.21	1.83 ± 1.9	NS
	After 12 months	1.2 ± 0.5	1.3 ± 0.9	NS
* p * *		<0.001	<0.001	
IIEF-15 score (*n*)	Before	11.42 ± 5.17	13.76 ± 6.25	0.001
	After 3 months	16.73 ± 2.28	15.17 ± 2.55	0.002
	After 6 months	17.29 ± 2.16	14.77 ± 2.33	0.001
	After 12 months	17.44 ± 1.88	15.13 ± 2.10	0.001
* p * *		<0.001	NS	

IIEF: International Index of Erectile Function; NS: not significant; VAS: visual analog scale; *p* *: *p* value within group before vs. 12 months after treatment.

## Data Availability

The original contributions presented in the study are included in the article. Further inquiries can be directed to the corresponding author.

## References

[1] Sharma K.L., Alom M., Trost L. (2020). The Etiology of Peyronie’s Disease: Pathogenesis and Genetic Contributions. Sex. Med. Rev..

[2] Ziegelmann M.J., Bajic P., Levine L.A. (2020). Peyronie’s disease: Contemporary evaluation and management. Int. J. Urol..

[3] Campbell J.D., Matti D., Abed H., Di Pierdominico A. (2022). Technological Advancements for Treating Erectile Dysfunction and Peyronie’s Disease. Urol. Clin. N. Am..

[4] Rosenberg J.E., Ergun O., Hwang E.C., Risk M.C., Jung J.H., Edwards M.E., Blair Y., Dahm P. (2023). Non-surgical therapies for Peyronie’s disease. Cochrane Database Syst. Rev..

[5] Zhang D.L., Chen Z., Wang F.X., Zhang J., Xie H., Wang Z.Y., Gu Y.B., Fu Q., Song L.J. (2019). Adding a vacuum erection device to regular use of Tadalafil improves penile rehabilitation after posterior urethroplasty. Asian J. Androl..

[6] Cai T., Capece M., Ceruti C., Tiscione D., Puglisi M., Verze P., Gontero P., Palmieri A. (2023). The Use of Vacuum Devices as Adjuvant Therapy before and after Penile Curvature Surgery in Patients Affected by La Peyronie’s Disease: Results from a Comparative Study. Clin. Pract..

[7] Campbel J., Alzubaidi R. (2017). Understanding the cellular basis and pathophysiology of Peyronie’s disease to optimize treatment for erectile dysfunction. Transl. Androl. Urol..

[8] Dell’Atti L. (2015). Tadalafil once daily and intralesional verapamil injection: A new therapeutic direction in Peyronie’s disease. Urol. Ann..

[9] Kovanecz I., Rambhatla A., Ferrini M.G., Vernet D., Sanchez S., Rajfer J., Gonzalez-Cadavid N. (2008). Chronic daily tadalafil prevents the corporal fibrosis and veno-occlusive dysfunction that occurs after cavernosal nerve resection. BJU Int..

[10] Gianazza S., Belladelli F., Leni R., Masci F., Rossi P., Gianesini G., Maggio P., Zaffuto E., Salonia A., Carcano G. (2023). Peyronie’s disease development and management in diabetic men. Andrology.

[11] Corona G., Cucinotta D., Di Lorenzo G., Ferlin A., Giagulli V.A., Gnessi L., Isidori A.M., Maiorino M.I., Miserendino P., Murrone A. (2023). The Italian Society of Andrology and Sexual Medicine (SIAMS), along with ten other Italian Scientific Societies, guidelines on the diagnosis and management of erectile dysfunction. J. Endocrinol. Investig..

[12] Dell’Atti L., Ronchi P. (2023). Low-intensity laser diode plus extracorporeal shock wave therapy: A new treatment strategy in the management of Peyronie’s disease. World J. Urol..

[13] Hauck E.W., Mueller U.O., Bschleipfer T., Schmelz H.U., Diemer T., Weidner W. (2004). Extracorporeal shock wave therapy for Peyronie’s disease: Exploratory meta-analysis of clinical trials. J. Urol..

[14] Hatzichristodoulou G., Meisner C., Gschwend J., Stenzl A., Lahme S. (2013). Extracorporeal shock wave therapy in Peyronie’s disease: Results of a placebo-controlled, prospective, randomized, single-blind study. J. Sex. Med..

[15] Shimpi R.K., Jain R.J. (2016). Role of extracorporeal shock wave therapy in management of Peyronie’s disease: A preliminary report. Urol. Ann..

[16] Vardi Y., Appel B., Kilchevsky A., Gruenwald I. (2012). Does low intensity extracorporeal shock wave therapy have a physiological effect on erectile function? Short-term results of a randomized, double-blind, sham controlled study. J. Urol..

[17] Gruenwald I., Appel B., Vardi Y. (2012). Low-intensity extracorporeal shock wave therapy—A novel effective treatment for erectile dysfunction in severe ED patients who respond poorly to PDE5 inhibitor therapy. J. Sex. Med..

[18] Palmieri A., Imbimbo C., Longo N., Fusco F., Verze P., Mangiapia F., Creta M., Mirone V. (2009). A first prospective, randomized, double-blind, placebo-controlled clinical trial evaluating extracorporeal shock wave therapy for the treatment of Peyronie’s disease. Eur. Urol..

[19] Raheem Amr A., Garaffa G., Raheem Tarek A., Dixon M., Kayes A., Christopher N., Ralph D. (2010). The role of vacuum pump therapy to mechanically straighten the penis in Peyronie’s disease. BJU Int..

[20] Mortensen J., Skov-Jeppesen S.M., Ladegaard P.B.J., Lund L. (2021). A Randomized, Single-Blinded Clinical Trial Evaluating the Effect of Extracorporeal Shockwave Treatment (ESWT) as Add-On Therapy to Vacuum Erectile Device on Peyronie’s Disease. Res. Rep. Urol..

[21] Chirca N., Streinu-Cercel A., Stefan M., Aurelian J., Persu C. (2023). A novel risk calculator to predict erectile dysfunction in HIV-positive men. J. Pers. Med..

[22] Park H.J., Park N.C., Kim T.N., Nam J.K., Moon D.G. (2017). Daily tadalafil therapy: A new treatment option for Peyronie’s disease?. Eur. Urol. Suppl..

[23] Porst H., Hell-Momeni K., Büttner H. (2009). Chronic PDE-5 inhibition in patients with erectile dysfunction: New treatment approach using once daily 2009. Urol. A.

[24] Palmieri A., Imbimbo C., Creta M., Verze P., Fusco F., Mirone V. (2012). Tadalafil once daily and extracorporeal shock wave therapy in the management of patients with Peyronie’s disease and erectile dysfunction: Results from a prospective randomized trial. Int. J. Androl..

[25] Rassweiler J.J., Scheitlin W., Goezen A.S., Radecke F. (2024). Long-term experiences with high-energy shock wave therapy in the management chronic phase Peyronie’s disease using two different electromagnetic lithotripters. World J. Urol..

